# Feasibility and Comparison of Resting Full-Cycle Ratio and Computed Tomography Fractional Flow Reserve in Patients with Severe Aortic Valve Stenosis

**DOI:** 10.3390/jcdd9040116

**Published:** 2022-04-14

**Authors:** Hendrik Wienemann, Marcel C. Langenbach, Victor Mauri, Maryam Banazadeh, Konstantin Klein, Christopher Hohmann, Samuel Lee, Isabel Breidert, Alexander Hof, Kaveh Eghbalzadeh, Elmar Kuhn, Marcel Halbach, David Maintz, Stephan Baldus, Alexander Bunck, Matti Adam

**Affiliations:** 1 Clinic III for Internal Medicine, Faculty of Medicine and University Hospital Cologne, University of Cologne, Kerpener Str. 61, 50937 Cologne, Germany; victor.mauri@uk-koeln.de (V.M.); maryam.b@hotmail.de (M.B.); christopher.hohmann@uk-koeln.de (C.H.); samuel.lee@uk-koeln.de (S.L.); isabel.breidert@uk-koeln.de (I.B.); alexander.hof@uk-koeln.de (A.H.); marcel.halbach@uk-koeln.de (M.H.); stephan.baldus@uk-koeln.de (S.B.); matti.adam@uk-koeln.de (M.A.); 2 Department of Radiology, Faculty of Medicine and University Hospital Cologne, University of Cologne, Kerpener Str. 61, 50937 Cologne, Germany; marcel.langenbach@uk-koeln.de (M.C.L.); konstantin.klein1@uk-koeln.de (K.K.); david.maintz@uk-koeln.de (D.M.); alexander.bunck@uk-koeln.de (A.B.); 3 Department of Cardiothoracic Surgery, Faculty of Medicine and University Hospital Cologne, University of Cologne, Kerpener Str. 61, 50937 Cologne, Germany; kaveh.eghbalzadeh@uk-koeln.de (K.E.); elmar.kuhn@uk-koeln.de (E.K.)

**Keywords:** coronary artery disease, computed tomography fractional flow reserve, invasive coronary angiography, resting full-cycle ratio, aortic valve stenosis

## Abstract

**Background**: Computed tomography derived Fractional Flow Reserve (CT-FFR) has been shown to decrease the referral rate for invasive coronary angiography (ICA). The purpose of the study was to evaluate the diagnostic performance of CT-FFR compared to hyperemia-free index Resting Full-cycle Ratio (RFR) in patients with relevant aortic stenosis (AS) and intermediate coronary stenosis. **Methods**: 41 patients with 46 coronary lesions underwent ICA with quantitative coronary angiography (QCA), pressure wire assessment and routine pre-transcatheter aortic valve replacement (TAVR) computed tomography (CT). CT-FFR analysis was performed using prototype on-site software. **Results:** RFR showed a significant correlation with CT-FFR (Pearson’s correlation, r = 0.632, *p* < 0.001). On a per-lesion basis, diagnostic accuracy, sensitivity, specificity, positive predictive value, and negative predictive value of CT-FFR were 82.6% (95% CI 68.6–92.2), 69.6% (95% CI 47.1–86.8), 95.7% (95% CI  78.1–99.9), 94.1% (95% CI 69.8–99.1), and 75.9% (95% CI 62.7–85.4), respectively. The optimal cutoff value of the CT-FFR for RFR ≤ 0.89 prediction was 0.815. The area under the receiver curve showed a larger area under the curve for CT-FFR (0.87; 95% CI 0.75–0.98) compared with CTA stenosis of ≥50% (0.54, 95% CI 0.38–0.71), CTA ≥ 70% (0.72, 95% CI 0.57–0.87) and QCA ≥ 50% (0.67, 95% CI 0.52–0.83). **Conclusions:** CT-FFR assessed by routine pre-TAVR CT is safe and feasible and shows a significant correlation with RFR in patients with AS. CT-FFR is superior to QCA ≥ 50%, CT ≥ 50% and CT ≥ 70% in assessing the hemodynamic relevance of intermediate coronary lesions. Thus, CT-FFR has the potential to guide revascularization in patients with AS.

## 1. Introduction

Transcatheter aortic valve replacement (TAVR) has become the standard treatment of severe aortic valve stenosis (AS) for elderly patients and those with an intermediate or high risk for surgery [1]. Computed tomography (CT) scan protocols for TAVR procedure planning vary between centers. In the majority, a dedicated coronary computed tomography angiography (cCTA) is not routinely performed. But AS is often accompanied by coronary artery disease (CAD), which strongly affects the one-year mortality of these patients [2]. Due to the high pre-test probability for CAD, these patients routinely undergo invasive coronary angiography (ICA) prior to TAVR to assess CAD and to perform revascularization by percutaneous intervention (PCI) in case of significant left main or proximal CAD according to current guidelines [1].

Recent studies report that pre-interventional CT might be useful to exclude relevant coronary artery disease and precede ICA [3,4]. However, routine cCTA alone is of limited value to exclude CAD in cases of severe calcification, leaving room for major improvements [5]. Also, the high false-positive rate of cCTA for obstructive CAD leads to unnecessary ICA [4].

Physiological assessment improves clinical outcomes over management based on angiography alone [6,7,8]. The combination of conventional pre-TAVR CTs with physiological assessment might provide incremental benefits in patients with AS. But measurement of fractional flow reserve (FFR) to assess the functional severity of intermediate coronary stenosis requires hyperemia, which is mostly induced by adenosine [9]. This can cause dyspnea, chest discomfort and severe arrhythmias, especially in the elderly population. Therefore, the development of resting physiological indices known as non-hyperemic pressure ratios (NHPRs) are of great importance. As one particularly relevant NHPR, the Resting Full Cycle Ratio (RFR) is calculated as the minimal distal pressure in relation to the aortic pressure during five entire cardiac cycles [10]. Yet, given the paucity of available data for FFR and NHPRs in patients with AS, conventional cut-off values for AS patients are still not established [11].

To date, CT-FFR analysis for clinical use is only available as a commercial off-site evaluation (HeartFlow Inc. (Redwood City, CA, USA) [12,13]. A recent study reported high diagnostic accuracy of CT-FFR in coronary CTA scans compared to invasive FFR in patients with AS [14].

A recent on-site prototype software developed by Siemens Healthineers (Erlangen, Germany) with different computational fluid dynamics calculations shortens the analysis time. This algorithm has been studied against invasive FFR and established CT-FFR algorithms with substantial diagnostic accuracy [15].

The diagnostic accuracy, clinical performance, and correlation of CT-FFR assessed by pre-TAVR CT in comparison with RFR in the special subset of patients with AS is yet unknown. Therefore, the objective of this study was to investigate the feasibility of CT-FFR based on a routine pre-TAVR CT compared to invasive RFR assessment in patients with aortic valve stenosis.

## 2. Materials and Methods

### 2.1. Study Subjects

This retrospective observational, longitudinal single-center study was conducted at the University Hospital Cologne between August 2015 and December 2019. Patients included in this analysis required TAVR assessment on a determined CT-system (Somatom Force, Siemens Healthineers, Erlangen, Germany) and a subsequent [within three months thereafter] invasive physiological lesion assessment due to at least one major intermediate coronary lesion (diameter stenosis 30–80%), determined visually by the treating physician. All interrogated vessels were analyzed in this study. Following these assessments, the appropriate patients were scheduled to undergo TAVR.

Exclusion criteria were ICA in another hospital, significant left main disease, prior coronary artery bypass graft surgery, chronic total occlusions, use of a different CT-system, CT scan after PCI, different CT scan protocol, or insufficient image quality. 

We analyzed demographic and clinical data on a per-patient level in case of multi-vessel disease. 

The study was approved by the local ethics committee and was conducted in accordance with the Declaration of Helsinki.

### 2.2. Acquisition and Analysis CT Datasets (CT-TAVR Image Reconstruction)

Data were acquired using a third-generation dual source 256-slice CT-system (Somatom Force, Siemens Healthineers, Erlangen, Germany). The protocol was in accordance with the Society of Cardiovascular Computed Tomography (SCCT) [16]. As a CT imaging protocol, a tube voltage of 100 kV with dose-modulation was set for a quality reference of 300 mAs. Collimation was 192 × 0.6 mm, pitch factor 3.2 with a rotation time of 0.25 s. Patients received 60 mL iodinated contrast medium (Imeron 400, Bracco Imaging S.p.A., Milan, Italy) using a power injector (Accutron CT, Metron, Saarbrücken, Germany) with a standardized injection protocol, administered via intravenous access in the right antecubital vein. This contained a bolus consisting of 60 mL ICM at an injection rate of 5 mL/s and a 40 mL saline chaser injected with 5 mL/s. We performed an ECG-gated high-pitch-scan (pitch 3.2) for analysis of the aortic valve, coronary vessels, and depiction of the access route (scan time: approximately 2 s). The threshold for automatic initiation of the standardized TAVR CT-protocol was 120 HU in the ascending aorta.

For the retrospective image analysis, we used a qualified on-site-software (syngo.via VB40A, Siemens Healthineers, Erlangen, Germany). Two radiologists with three and five years’ experience (K.K., M.C.L.) in cardiac imaging performed visual and semi-automated assessments of the coronary vessels in consensus. The readers were blinded to RFR results. All coronary vessels with a diameter of >1.5 mm were analyzed. 

Image quality was analyzed using a 5-point Likert-scale: 1—non diagnostic, 2—diagnostic despite impairment by image noise, artifacts, and/or low contrast opacification, 3—moderate image noise with sufficient intraluminal visibility, artifacts may be present, 4—good vessel contrast in the absence of major artifacts, low image noise, 5—excellent, no diagnostic limitations. The mean artifact score was defined using a 3-point Liker-scale: 0—excellent, no artifacts to 3—non diagnostic, severe artifacts, analogue for the calcifications score: 0—absent to 3—severe. 

Coronary arteries were analyzed by segment visually and semi-automated for the degree of stenosis, the luminal diameter, lesion length, and plaque characteristics (calcified, non-calcified, mixed).

### 2.3. CT-FFR Analysis

The same two radiologists performed the post-processing of the CT dataset using a validated on-site software prototype (cFFR version 3.0, Siemens Healthineers, Erlangen, Germany; currently not commercially available). CT-FFR calculation was performed after the semi-automated definition of the vessels and the lumen. The software generated an anatomic color-coded 3D coronary artery tree model. CT-FFR values were measured 1–2 cm distal to the most severe stenosis in the interrogated vessel. Ischemic obstructive CAD was defined with a lesion-specific CT-FFR value of ≤0.80.

### 2.4. Invasive Coronary Angiogram and Resting Full Cycle Ratio Measurement

Coronary angiography was performed in accordance with standard clinical practice and radial first approach when feasible, otherwise femoral access was used. Intracoronary pressure was measured with the PressureWire™ X Guidewire (Abbott Vascular Inc., Santa Clara, CA, USA). FFR measurements were anonymously transferred to an independent core laboratory (CoroLab; Coroventis Research AB, Uppsala, Sweden) with a completely automated offline software algorithm for calculating RFR. In 17 lesions RFR was directly measured at an RFR-Workstation (Quantien System v.1.12; Abbott Vascular). The threshold for hemodynamically significant stenosis (RFR ≤ 0.89) was defined according to current recommendations. Diameter stenosis was assessed by two-dimensional quantitative coronary angiogram (QCA) analysis using the QAngio XA software package (Medis Medical Imaging Systems, Leiden, The Netherlands).

### 2.5. Statistical Analysis

Continuous variables are presented as mean ± standard deviation or median with interquartile range, while categorical variables are reported as frequencies and percentages. The Pearson coefficient was applied to illustrate correlations between values. Comparison of categorical data were made using chi-square statistics. Cut-off values were calculated with the Youden-Index and graphically controlled based on ROC curves to determine the diagnostic value (area under the curve [AUC] and accuracy) for each index with respect to RFR ≤  0.89, indicating hemodynamic relevance. Diagnostic accuracy was calculated as the summation of true positive and true negative divided by the total population for each threshold. Bland-Altman plots, sensitivity, specificity, negative, and positive predictive value were used to examine the diagnostic agreement and 95% limits of agreement. Two-tailed *p*-values < 0.05 were defined as statistically significant. A statistical analysis was conducted in SPSS Statistics, version 27 (IBM, Armonk, NY, USA).

## 3. Results

### 3.1. Baseline Characteristics

Forty-six vessels in 41 patients were analyzed. The mean patient age was 80.9 ± 6.2 years, mean EuroSCORE II was 3.5 ± 2.7 %, and 46 % were female. Diabetes mellitus was present in 14 patients (34.1%). The mean aortic pressure gradient and aortic valve area were 48.1 ± 14.4 mmHg and 0.74 ± 0.17 cm^2^, respectively. Clinical characteristics of the study cohort are summarized in Table 1. 

The left anterior descending was investigated most frequently (69.6%). Mean QCA %DS was 48.6 ± 9.2 (Table 2). 23 (50%) of lesions had an invasive RFR ≤ 0.89 as the reference standard.

### 3.2. Scan Demographics and CT-Assessment

CTs were performed with a mean heart rate of 77.2 ± 17.5 beats per minute. Mean dose length product (DLP) was 290.64 ± 126.57 (mGy*cm). Qualitative assessment of CT-imaging per patient was as follows: moderate image noise with sufficient intraluminal visibility (n = 17, 41.5%), good vessel contrast with the possibility of minor artifacts and a low image noise (n = 14, 34.1%). After assessment of imaging artifacts, most CT scans showed only minor artifacts (n = 26, 63.4%), and there were no cases with severe artifacts. The calcification score was moderate in 22 patients (53.27%). Details are displayed on a per-vessel basis in the supplementary data online, Table S1. The mean time per patient needed for CT-FFR analysis was 23 ± 8.3 min. Plaque characteristics were mainly described as calcified. Mean RFR and CT-FFR were 0.89 ± 0.08 and 0.85 ± 0.08, respectively. CT-FFR classified 17 lesions as ischemic (CT-FFR ≤ 0.80). The number of ischemic lesions as classified by CT-FFR ≤ 0.80 or RFR ≤ 0.89 were similar for both methods (n = 17, 36.9% vs. n = 23, 50%, respectively; *p* = 0.21).

### 3.3. Correlation between CT-FFR and RFR and Anatomic Grading

**Figure 1:**
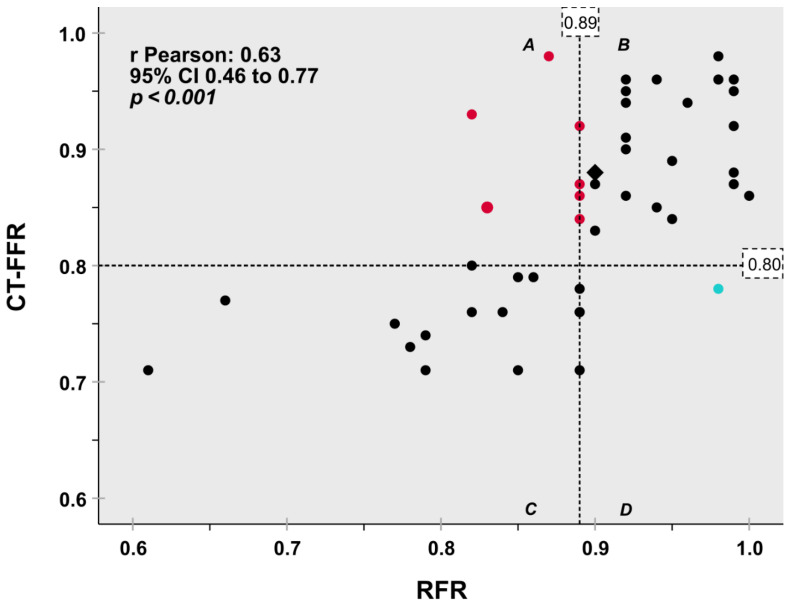
Scatter plot diagram for classification agreement of the hemodynamic relevance of coronary artery stenosis on a per lesion basis depicts good agreement between CT-FFR with invasive RFR as the reference standard. Dashed lines indicate the 0.80 CT-FFR and 0.89 RFR cut-off values. 82.6% concordant classification between CT-FFR and RFR (quadrants B + C, black symbols) was observed. One lesion [2.2%] was false positive classified by CT-FFR (turquoise symbol, quadrant D). Seven lesions [15.2%] were false negative (red symbols, quadrant A). Two assessments yielded identical values (Rhombus). CT-FFR fractional flow reserve based on computed tomography angiography, RFR Resting full cycle ratio.

A modest correlation coefficient between CT-FFR and RFR was shown (r = 0.632; 95% CI: 0.46–0.77, *p* < 0.001) (Figure 1).

**Figure 2:**
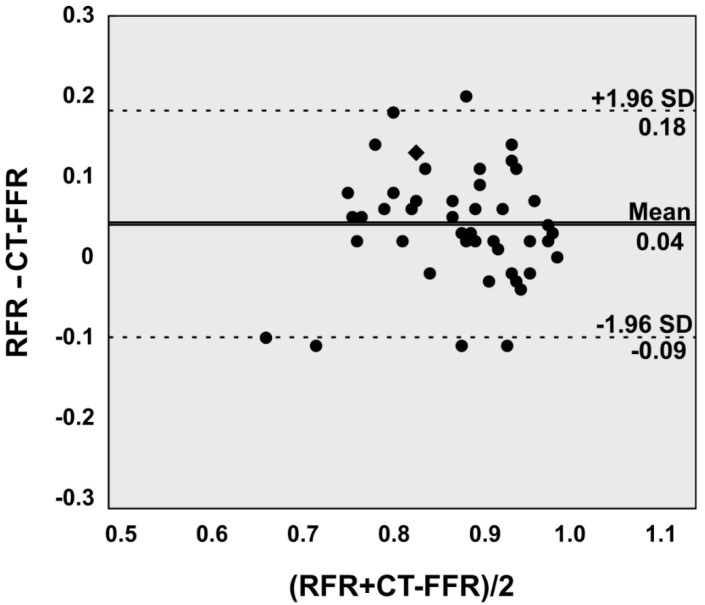
Bland-Altman plot illustrates good agreement between Resting full cycle ratio (RFR) and computed tomography angiography based fractional flow reserve (CT-FFR) on a per lesion level. Two assessments yielded identical values (Rhombus).

This is mirrored by the agreement (mean difference and root mean squared deviation of 0.04 ± 0.07; 95% limits of agreement −0.09 to 0.18) between CT-FFR and RFR as displayed graphically by Bland-Altman plots (Figure 2). 

### 3.4. Diagnostic Accuracy of CT-FFR and CT

Based on a per vessel level analysis, the classification agreement, sensitivity, specificity, positive predictive value, negative predictive value, and diagnostic accuracy of CT-FFR ≤ 0.8, CTA ≥ 50%, CTA ≥ 70%, QCA (stenosis ≥ 50%) to identify ischemic lesions defined as RFR ≤ 0.89 are shown in Table 3. The sensitivity and specificity of CT-FFR to determine the RFR-based functional stenosis severity were 69.6% and 95.7%, respectively. The diagnostic accuracy of %DS by QCA or CT in assessing the functional stenosis severity were inferior to CT-FFR.

**Figure 3:**
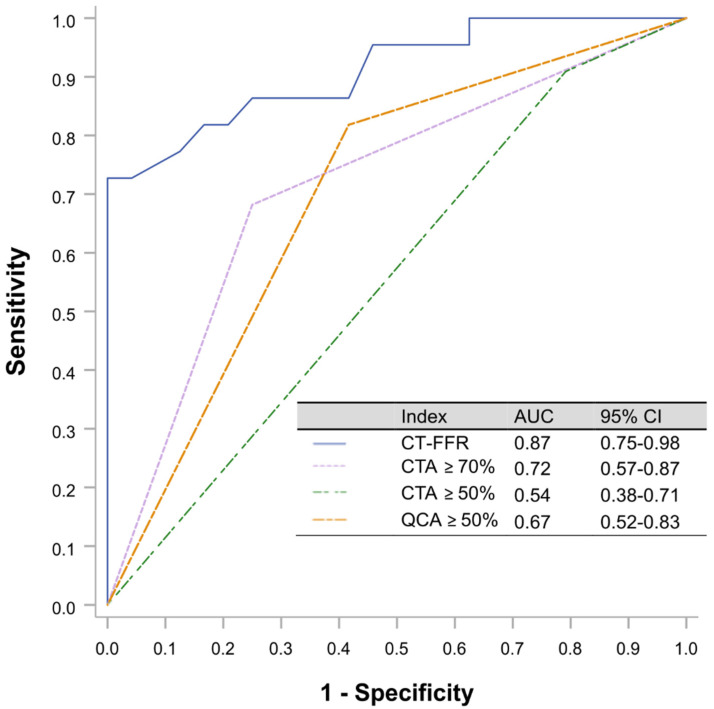
ROC curves were calculated using RFR as the reference gold standard. The threshold cut-off for RFR was ≤0.89. The optimal CT-FFR cutoff value for predicting RFR 0.89 was 0.815 (sensitivity 69.6%, specificity 95.7%, Youden index 0.65). CTA, computed tomography angiography; CT-FFR, coronary computed tomography angiography based fractional flow reserve; RFR, resting full cycle Ratio; ROC, receiver operating characteristic curves.

The area under the curve (AUC) to predict RFR ≤ 0.89 on a per vessel level analysis by CT-FFR using RFR as the reference standard were 0.87 (95% CI 0.75–0.98) compared with that for CTA ≥ 50% 0.54 (95% CI 0.38–0.71), by CTA ≥ 70% 0.72 (95% CI 0.57–0.87) and QCA ≥ 50% 0.67 (95% CI 0.52–0.83). ROC analysis identified CT-FFR ≤ 0.815 as the optimal binary cut-off to predict an RFR ≤ 0.89 (Figure 3). 

**Figure 4:**
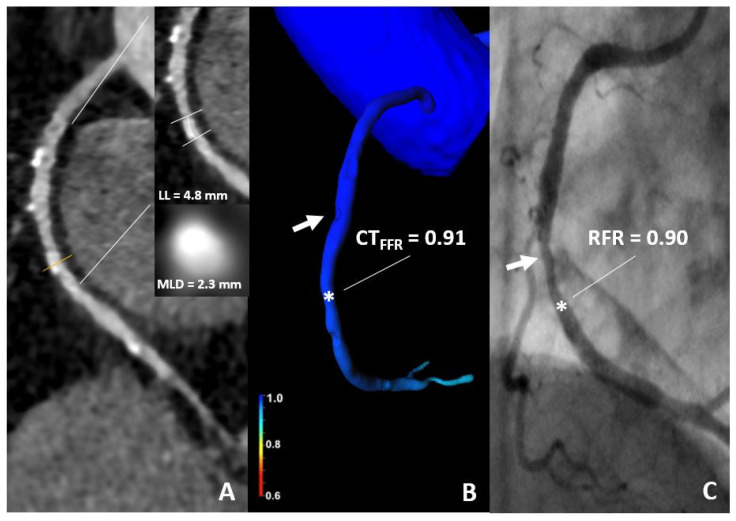
86-year-old woman. (**A**) CT demonstrates a 60% stenosis of the mid segment of the RCA. (**B**) The CT-FFR value was 0.91 (star). (**C**) An ICA with RFR value of 0.90 (star) shows a non-significant stenosis. CT, computed tomography; CT-FFR, coronary computed tomography angiography based fractional flow reserve; RFR, resting full cycle Ratio; LL, lesion length; MLD, minimal luminal diameter.

**Figure 5:**
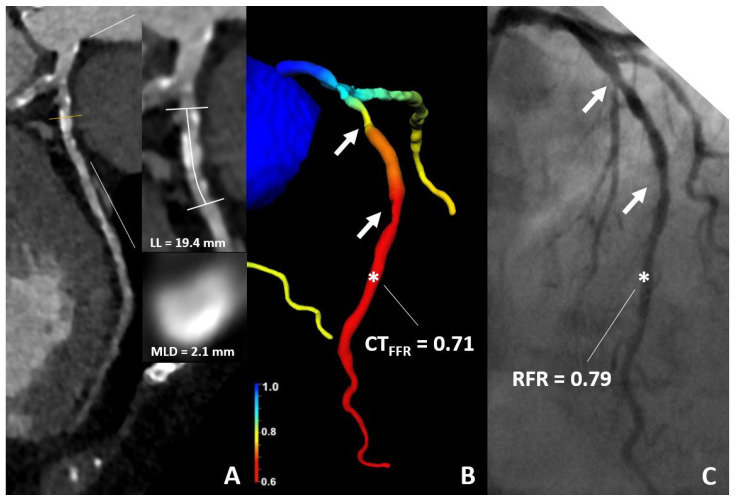
76-year-old woman. (**A**) CT demonstrates sequential 50% stenoses of the LAD. (**B**) The CT-FFR value was of 0.71 (star). (**C**) ICA confirmed ischemia with an RFR-value of 0.79 (star); RFR Cut-Off ≤ 0.89. CT, computed tomography; CT-FFR, coronary computed tomography angiography based fractional flow reserve; RFR, resting full cycle Ratio; LL, lesion length; MLD, minimal luminal diameter.

Case examples are illustrated in Figure 4 and Figure 5.

## 4. Discussion

According to current guidelines, revascularization in significant obstructive proximal coronary arteries is recommended in patients undergoing TAVR [1,17]. For patients presenting with a stenosis between 40–90% by visual assessment, invasive pressure measurement is recommended (Class Ia) [17]. However, in the major physiological trials, patients with AS were not included [18,19].

The present study sought to investigate the applicability of CT-FFR in pre-TAVR CT scans. Furthermore, we compared CT-FFR to invasive pressure wire measurements with RFR in patients with AS. 

The main findings of this study are as follows:

(i) The utilization of routine TAVR-CTs for the analysis of CT-FFR is technically feasible and allows for the assessment of CAD in patients with AS even without a specific coronary imaging protocol.
(ii) Compared with anatomical assessment, either by CT or QCA, functional assessment utilizing CT-FFR is superior in predicting ischemic lesions as classified by invasive physiological assessment with RFR.

In the present study we exclusively evaluated AS patients who were eligible in the presence of intermediate coronary artery stenosis. To overcome the limits of usage incurred by FFR, NHPRs may become the standard in physiological assessment for patients with AS, but optimal cut-off values are currently being discussed [11,20]. The instantaneous wave-free ratio (iFR) showed a good correlation with FFR in patients with AS [20]. However, in comparison to FFR, iFR may classify stenosis severity differently in patients with aortic stenosis [21]. The resting coronary flow in patients with AS is increased, leading to a smaller amplitude between hyperemic and resting flow, which may contribute to these technical differences [22]. 

We applied RFR as NHPRs reference standard. RFR enables assessment during the diastolic and systolic cardiac phase [10]. Physiological assessment by RFR might lead to higher detection of ischemia compared to the diastolic pressure index [23]. The structural alterations in severe aortic valve stenosis lead to coronary microcirculatory dysfunction and increased minimal microvascular resistance [22,24]. This might translate into pathological augmented rise in early systolic deceleration [25]. Regarding these mechanistic considerations, RFR might be the ideal NHPR in patients with AS. A recent small substudy of the Notion-3 trial found that RFR is better than FFR to detect physiologically nonsignificant stenoses before TAVR [26]. 

In general, CT-guided deferral from ICA appears to be safe in patients with no obstructive coronary artery disease [27]. Regarding anatomical parameters for CAD severity in our collective with AS, our findings confirm that the definition of relevant CT stenosis ≥ 70% increases the specificity for detection of relevant CAD. As expected, this results in lower sensitivity compared to CT stenosis definition ≥ 50%, which is in line with a previous report [28]. In our study, the overall diagnostic accuracy to detect functional significant stenosis was increased from 54.4% to 71.7%. A prior study yielded a similar diagnostic accuracy for CT compared to QCA for the identification and exclusion of lesion specific ischemia [29]. Nevertheless, lesion classification based on ‘percent diameter stenosis’ has limited value in correctly detecting ischemia-producing lesions based on functional evaluation [13]. 

The quantitative flow ratio (QFR) obtained from 3-dimensional QCA facilitates on site physiological assessment in intermediate stenoses without the need for pressure-wires and hyperemic drugs [30]. Multicenter studies, including larger consecutive patient cohorts, have validated the benefit of revascularization guidance compared to angiography [31,32,33]. In addition, QFR depicted a higher diagnostic accuracy compared to angiography to identify functionally significant coronary lesions in AS patients [34]. 

Patients with AS are more often categorized in increased risk categories with a higher burden of vascular and coronary calcification. Therefore, the diagnostic capability of CT based visual assessment in ruling out a CAD is limited and even more dependent on operator and center experience [5,35]. 

Hence, non-invasive functional lesion classification via CT-FFR might help increase diagnostic accuracy [36,37]. Studies have shown a good correlation between invasive FFR and CT-FFR [13,38]. CT-FFR has only been validated for specific heart scans using an additional cCTA scan and is limited by high costs and offline use [13,14,39].

Nevertheless, utilizing a pre-TAVR CT protocol with high-pitch acquisition without a dedicated scan of the coronary artery tree, thus without a specific coronary CTA, allowed us to gather valid data. Here, state of the art CT computed tomography systems with a high temporal and spatial resolution might be an important prerequisite to optimize quality.

With an AUC of 0.87 and diagnostic accuracy of 82.6% in our study, CT-FFR based on routinely performed TAVR-CT may reduce the number of ICA in patients with AS. In our cohort, diagnostic yield was excellent and similar to the findings of Michail et al. [14].

The cut-off 0.815 for CT-FFR by ROC curve analysis has been very close, with recommended CT-FFR cut-off values for non-AS patients set to 0.80, which is based on data from various large trials [13,38]. To date, FFR-CT analysis by HeartFlow Inc. is commercially available for clinical use. Our results are in line with another report using a comparable algorithm [40]. Nevertheless, the applied on-site Siemens CT-FFR software is a research software prototype that is currently not commercially available for clinical use. 

As for safety concerns, CT-FFR classified only one lesion (LCX) as false positive on a per-vessel analysis (2.2%). In comparison, the results of Michail et al. showed a false positive CT-FFR classification of 13% using invasive FFR as the reference standard [14].

The comparison of RFR and CT-FFR has only been validated in patients with chronic coronary syndrome. Here, an excellent diagnostic accuracy of 93% could be shown [40]. RFR appears to correlate significantly with CT-FFR in our study (Pearson’s correlation of 0.632). Between iFR and CT-FFR, previous studies report correlation coefficients between 0.62–0.82 [41,42].

In conclusion, the application of CT-FFR on routine pre-TAVR CTs is technically possible and safe, but knowledge of the limitations and indications for referral to ICA are important. Most importantly, our analyses have been utilized without additional scan time, with no extra medication, and with no change in the TAVR CT scan protocol. 

If these findings are confirmed in future prospective trials, routine pre-TAVR CT evaluation in patients with aortic stenosis might bear significant potential for functional coronary measurements. This might help to define indications for ICA in these patients more precisely.

### Limitations

This is an observational study limited by its retrospective design. In contrast to previous studies, we included only vessels with physiological assessment at the operator’s discretion. Therefore, no statement can be conducted for severely stenosed (>80%) or non-stenosed vessels since they were not interrogated with invasive wire assessment. 

Moreover, we report data from a single center with a limited number of patients due to the pilot character, raising the possibility of selection bias. As discussed, for TAVR assessment, patients received a high-pitch CT-scan without a specific cCTA-scan. This may have limited the diagnostic value. Furthermore, agreement between CT-FFR and FFR or RFR after TAVR was not evaluated, and in this respect the data on coronary physiology is limited. 

## 5. Conclusions

As compared to invasive assessment by RFR, CT-FFR has good diagnostic accuracy and is superior to visual-only assessment of coronary arteries in CT and QCA in AS patients scheduled for TAVR. The study shows that CT-FFR has the potential to detect the majority of patients with hemodynamically relevant coronary artery disease. Taken together, standard pre TAVR-CT allows subsequent anatomical and functional coronary assessment in patients with severe aortic stenosis. It might therefore emerge as a gatekeeper for ICA in this cohort of patients.

## Figures and Tables

**Table 1:** Patient characteristics (n = 41).

**Demographics**	
Age (years)	80.9 ± 6.2
Body-mass index	26.6 ± 5.1
Body-surface area	1.8 ± 0.2
Female sex	19 (46.3)
**Cardiovascular risk factors and concomitant diseases**	
Hypertension	34 (82.9)
Dyslipidemia	23 (56.1)
Diabetes mellitus	14 (34.1)
Peripheral artery disease or extracardiac arteriopathy	7 (17.1)
Prior myocardial infarction	1 (2.4)
Atrial fibrillation	8 (19.5)
Chronic obstructive lung disease	5 (12.2)
**Baseline blood values**	
Serum creatinine—mg/dL	1.1 ± 0.4
Hemoglobin—g/dL	12.7 ± 1.59
**Echocardiography**	
Ejection fraction	
Normal (>50%)	38 (92.7)
Mild dysfunction (41–50%)	3 (7.3)
Aortic valve mean gradient—mmHg	48.1 ± 14.4
Aortic valve maximum gradient—mmHg	77.5 ± 20.2
Peak aortic jet velocity—cm/s	431.6 ± 63.0
Aortic valve area—cm^2^	0.74 ± 0.17
**Risk Scores**	
LogEuroSCORE I—%	14.3 ± 8.8
EuroSCORE II—%	3.5 ± 2.7
Values are mean ± SD or n (%)	

**Table 2:** Lesion characteristics and physiological assessments (n = 46).

Lesion Characteristics and Physiological Assessments (n = 46)	
Prior revascularisation in any vessel	3 (6.5)
Prior stents in examined vessel	0
Measured vessel location territory	
Left anterior descending	32 (69.6)
Left circumflex artery	8 (17.4)
Ramus intermedius	1 (2.2)
Right coronary artery	5 (10.9)
Multivessel disease (n = 41)	19 (46.3)
SYNTAX Score (n = 41)	11.2 ± 6.2
Quantitative coronary angiography	
Diameter stenosis, %	48.6 ± 9.2
Cardiac computed tomography angiography	
Diameter stenosis, %	
20–49%	4 (8.7)
50–69%	29 (63.0)
70–90%	13 (28.3)
Functional indexes	
Resting full-cycle ratio	
Left anterior descending	0.87 ± 0.08
	0.89 (0.84–0.92)
Left circumflex artery	0.92 ± 0.06
	0.94 (0.86–0.98)
Ramus intermedius	0.94
Right coronary artery	0.91 ± 0.08
	0.92 (0.82–0.99)
CT-Fractional Flow Reserve	
Left anterior descending	0.84 ± 0.08
	0.85 (0.76–0.91)
Left circumflex artery	0.84 ± 0.1
	0.82 (0.76–0.94)
Ramus intermedius	0.85
Right coronary artery	0.88 ± 0.06
	0.87 (0.83–0.93)

Values are mean ± SD, median (IQR, 25th–75th percentiles), or n (%).

**Table 3:** Diagnostic performance of CT-FFR, CTA and QCA to identify RFR ≤ 0.89 on a per vessel basis.

	CT-FFR ≤ 0.80	CTA ≥ 50%	CTA ≥ 70%	QCA ≥ 50%
% Sensitivity	69.6 (47.1−86.8)	86.9 (66.4−97.2)	65.2 (42.7−83.6)	78.3 (56.3−92.5)
% Specificity	95.7 (78.1−99.9)	21.7 (7.5−43.7)	78.3 (56.3−92.5)	56.4 (34.5−76.7)
Positive Likelihood Ratio	16.0 (2.3−110.9)	1.1 (0.9−1.5)	3.0 (1.3−6.9)	1.8 (1.1−3.0)
Negative Likelihood Ratio	0.32 (0.17−0.59)	0.60 (0.16−2.22)	0.44 (0.24−0.81)	0.38 (0.16−0.90)
% Positive predictive value	94.1 (69.8−99.1)	52.6 (45.9−59.2)	75 (56.7−87.3)	64.3 (51.9−75)
% Negative predictive value	75.9 (62.7−85.4)	62.5 (31−86.1)	69.2 (55.3−80.4)	72.2 (52.5−85.9)
% Accuracy	82.6 (68.6−92.2)	54.4 (39−69.1)	71.7 (56.5−84.0)	67.4 (51.9−80.5)

Values are n (%) for classification agreement as categorical concordance, and % (95% CI) for all other parameters. CT-FFR, coronary computed tomography angiography based fractional flow reserve; QCA, quantitative coronary angiography, RFR, resting full cycle Ratio; ROC, receiver operating characteristic curves.

## Data Availability

The individual patient data that support the findings of this study are available on reasonable request to the corresponding author.

## Supplementary Materials

The following supporting information can be downloaded at: https://www.mdpi.com/article/10.3390/jcdd9040116/s1, Table S1: CT-Imaging and scan details per vessel.
