# Supplementary material for: Feasibility and Comparison of Resting Full-Cycle Ratio and Computed Tomography Fractional Flow Reserve in Patients with Severe Aortic Valve Stenosis
Source: J Cardiovasc Dev Dis. 2022 Apr 14;9(4):116. doi: 10.3390/jcdd9040116 (PMC9030550; doi:10.3390/jcdd9040116)
Supplement: Supplementary file 1 [file jcdd-09-00116-s001.zip › jcdd-1662445-supplementary.pdf]

Supplementary

Table S1. CT-Imaging and scan details per vessel.

Image Quality	
diagnostic despite impairment by image noise, artifacts, and/or low contrast opacification	6 (13)
moderate image noise with sufficient intraluminal visibility, artifacts may be present	20 (43.5)
good vessel contrast in the absence of major artifacts, low image noise	16 (34.8)
excellent, no diagnostic limitations	4 (8.7)
Artifact Score	
excellent, no artifacts	2 (4.3)
good	27 (58.7)
limited	17 (37.0)
Coronary Calcification	
mild	7 (15.2)
moderate	24 (52.2)
severe	15 (32.6)

Values are n (%)
